# Health Tract, No. 9

**Published:** 1860-02

**Authors:** 


### HEALTH TRACT, NO. 9.

### USES OF ICE.

In health no one ought to drink ice-water, for it has occasioned fatal inflammations of the stomach and bowels, and sometimes sudden death. The temptation to drink it is very great in summer; to use it at all with any safety the person should take but a single swallow at a time, take the glass from the lips for half a minute, and then another swallow, and so on. It will be found that in this way it becomes disagreeable after a few mouthfuls.

On the other hand, ice itself may be taken as freely as possible, not only without injury, but with the most striking advantage in dangerous forms of disease. If broken in sizes of a pea or bean, and swallowed as freely as practicable, without much chewing or crushing between the teeth, it will often be efficient in checking various kinds of diarrhœa, and has cured violent cases of Asiatic cholera.

A kind of cushion of powdered ice kept to the entire scalp, has allayed violent inflammations of the brain, and arrested fearful convulsions induced by too much blood there.

In croup, water, as cold as ice can make it, applied freely to the throat, neck, and chest, with a sponge or cloth, very often affords an almost miraculous relief, and if this be followed by drinking copiously of the same ice-cold element, the wetted parts wiped dry, and the child be wrapped up well in the bed-clothes, it falls into a delightful and life-giving slumber.

All inflammations, internal or external, are promptly subdued by the application of ice or ice-water, because it is converted into steam and rapidly conveys away the extra heat, and also diminishes the quantity of blood in the vessels of the part.

A piece of ice laid on the wrist will often arrest violent bleeding of the nose.

To drink any ice-cold liquid at meals retards digestion, chills the body, and has been known to induce the most dangerous internal congestions.

Refrigerators, constructed on the plan of Bartlett's, are as philosophical as they are healthful, for the ice does not come in contact with the water or other contents, yet keeps them all nearly ice cold.

If ice is put in milk or on butter, and these are not used at the time, they lose their freshness and become sour and stale, for the essential nature of both is changed, when once frozen and then thawed.

